# Impact of different surface conditioning protocols on fracture resistance of monolithic zirconia vonlay restorations

**DOI:** 10.1186/s12903-026-10032-y

**Published:** 2026-11-13

**Authors:** Ahmed Mahmoud EL-Zagzog, Mohamed Abd El-Rahman El-Layeh, Shaimaa Ahmed Abo El-Farag

**Affiliations:** 1 Department of Fixed Prosthodontics, Faculty of Dentistry, Mansoura University, Mansoura, Egypt; 2 Department of Fixed Prosthodontics, Faculty of Dentistry, Mansoura and Mansoura National University (MNU), Mansoura, Egypt

**Keywords:** Zirconia, Surface treatment, Laser, Hot chemical etching, Fracture resistance

## Abstract

**Background:**

The purpose of this study was to evaluate and compare the influence of three different surface conditioning protocols on the fracture resistance of maxillary premolars restored with monolithic multilayer zirconia vonlay restorations fabricated from two zirconia materials.

**Methods:**

Forty-eight maxillary premolars were prepared for zirconia vonlay restorations and allocated to two groups (*n* = 24) according to zirconia type: Group I (C): Cercon xt ML, and Group II (K): KATANA YML. Each group was further divided into three subgroups (*n* = 8) based on surface treatment: subgroup (A): airborne particle abrasion; subgroup (L): Er: YAG laser; and subgroup (E): hot chemical etching. Following cementation, all specimens were subjected to 10,000 thermocycles. Axial loading was applied using a universal testing machine. Data were analyzed using two-way ANOVA and Bonferroni’s post-hoc test, with one-way ANOVA conducted for each factor.

**Results:**

Two-way ANOVA demonstrated that the surface treatment had a significant effect on fracture resistance (*P* < 0.0001), whereas the main effect of zirconia material was not statistically significant (*P* = 0.229). The hot chemical etching KATANA YML subgroup recorded the highest mean values, 1476.25 (177.33) N, while the airborne particle abrasion and Cercon xt ML subgroup showed the lowest mean values, 1053.33 (172.78) N.

**Conclusion:**

The fracture resistance values of both zirconia materials were within the accepted range for clinical application. Hot chemical etching resulted in significantly higher fracture resistance than airborne particle abrasion and Er: YAG laser treatment, while no significant difference was found between the two zirconia materials.

## Background

The development of minimally invasive treatment approaches has been driven by the rising incidence of significant tooth loss due to caries and bio-corrosive defects, especially in younger populations [1]. Occlusal veneers are now considered the preferred option for minimally invasive posterior dental rehabilitation [2]. When caries involves the buccal and cervical regions, clinicians typically select either a complete coverage option or a partial occlusal restoration combined with a Class V composite filling. Although Class V fillings tend to have a reduced lifespan, complete coverage preparations result in unnecessary removal of healthy tooth structure [3]. Partial-coverage restorations offer a less invasive alternative by covering the occlusal, labial, and, when indicated, proximal surfaces [4, 5]. 

Yttrium-stabilized zirconia is distinguished among dental ceramics by its superior mechanical and physical properties [6, 7]. The phase transition in zirconia increases the material volume in the vicinity of a propagating fracture, thereby restricting further fracture propagation [8]. Increased yttria concentration for aesthetic purposes compromises material integrity and leads to premature failure of zirconia restorations [9]. Recently introduced zirconia materials contain varying concentrations of yttria, including 3Y, 4Y, and 5Y compositions. In multilayered zirconia, the yttria content may vary across the cervical and incisal/occlusal regions [10]. Each zirconia material has a different composition, shape, and surface characteristics, which can affect surface treatments and bond strength [11]. 

Adequate adhesion is critical to maintaining the structural integrity and durability of zirconia restorations under masticatory forces. Improved adhesion increases retention, marginal adaptation, and strength, especially when mechanical retention is limited [12–14]. Adhesively bonded all-ceramic restorations are more fracture-resistant than conventionally cemented ones. The luting process significantly contributes to this enhanced performance [15]. 

Zirconia adhesion is limited by its chemically inert nature, its dense, uniform post-sintering structure, and its lack of intrinsic glass content, which makes traditional hydrofluoric acid (HF) etching ineffective [16]. To improve the bond strength between resin cement and zirconia restorations, both micromechanical and chemical methods have been proposed. These include mechanical treatments, chemical treatments, laser applications, coupling agents, and silicon coatings [17–19]. 

Airborne particle abrasion is a widely used technique for improving the bond between resins and zirconia ceramics, ensuring a stronger, more reliable bond [20]. Numerous studies revealed that the abrasion technique has a role in boosting the strength of the adhesion between resin cements and zirconia restorations. Airborne particle abrasion significantly increases zirconia surface roughness and surface energy, and improves wettability for better bonding between resin and cement [21]. 

However, this procedure may modify the zirconia microstructure and induce phase transformations, potentially compromising its mechanical properties. The magnitude of these effects is determined by airborne particle abrasion parameters, including air pressure, application distance, angle, alumina particle size, and exposure duration. Additionally, the yttria concentration in zirconia affects the tetragonal-to-monoclinic phase transformation. Such alterations can diminish zirconia’s long-term stability by generating microcracks [22–27]. 

Kim HK et al. (2021) [28] investigated the influence of different Al_2_O_3_ particle sizes on three dental zirconia grades. The study identified a phase transformation from tetragonal to monoclinic in traditional zirconia, whereas highly translucent zirconia showed increased susceptibility to surface damage under applied stress.

Various lasers, including carbon dioxide (CO_2_), femtosecond, erbium-chromium-doped yttrium scandium gallium garnet (Er, Cr: YSGG), neodymium-doped yttrium aluminum garnet (Nd: YAG), and erbium-doped yttrium aluminum garnet (Er: YAG), have been utilized to improve zirconia bonding to tooth structure. Optimization of laser parameters is essential to maximize bond strength and surface roughness while preserving zirconia’s mechanical properties [29–32]. 

The Er: YAG laser alters several zirconia surface properties by increasing wettability and surface energy, causing a significant decrease in the average contact angle of liquids on the surface. Also, it increases surface roughness, resulting in a non-uniform topography characterized by higher peaks and wider valleys, especially in highly translucent zirconia. Er: YAG laser causes minimal to superficial tetragonal-to-monoclinic phase transformation in zirconia. Also, Nd: YAG laser treatment showed no phase transformation on the thermally affected surface, despite creating microcracks due to high temperature [33, 34]. 

In recent years, several chemical etching solutions have been evaluated for zirconia conditioning. These include Zeta etching solution; different concentrations of HF acid at various temperatures and durations; combinations such as H_2_SO_4_/NH_4_)_2_SO_4_, HF/HNO_3_, and H_2_SO_4_/HF/HNO_3_; and elevated-temperature etching with low concentrations of HF acid [35–38]. Hot chemical etching with a low concentration of hydrofluoric acid has been reported to significantly improve shear bond strength by creating a homogeneous, finely roughened surface with a uniform nanoporous structure, allowing cement to penetrate the surface more readily and establish microlocking between zirconia and the resin. Heat application makes the acidic solution more prone to ionization, accelerating the removal of surface particles. Minor monoclinic phase transformation occurs during hot chemical etching, presumed to be caused by structural deformation from heat treatment during the etching process [36]. 

Kang YJ et al. (2020) [36] found that hot chemical etching treatment with low concentrations of HF produced higher flexural and shear bond strengths than no surface treatment, abrasion, etching, or abrasion-etching groups, both before and after thermal cycling.

Recent advancements utilize reagents such as concentrated hydrochloric acid, ferric chloride, and methanol for prolonged durations, typically up to 60 min, in conjunction with heat treatment. Commercially available products, including Zircos-E solution and the Zirconia Etchant Cloud System, have demonstrated enhanced bond strength. These methods aim to improve bonding efficacy while maintaining surface integrity and preserving the mechanical properties of translucent zirconia [36, 39–41]. 

The present study evaluated and compared the effects of three surface-treatment methods on the fracture resistance of two types of monolithic multilayer zirconia partial-coverage restorations in maxillary first premolars. The null hypothesis of the study was that there would be no significant difference in the fracture resistance of maxillary first premolars restored with monolithic multilayer zirconia vonlay restorations subjected to different surface treatment methods.

## Materials and methods

### Materials

The study protocol was approved by the ethical committee of the Faculty of Dentistry, Mansoura University, with approval number M04011122. Materials used in this study are shown in Table 1.

**Table 1:** Showing the materials tested in this study

Product name	Batch Number	Composition	Manufacturer
KATANA zirconia YML (A2/D98.5 mm/T14mm)	EJHGY	Zirconium dioxide (ZrO_2_) & Hafnium oxide (HfO_2_) 89–96% Yttrium oxide (Y_2_O_3_) 5–10% Other oxides and pigments 0–2%.	Kuraray Noritake, Japan
Cercon xt ML (A2/D98.5 mm/T14mm)	18,047,015	ZrO_2_ (> 86 wt%) HfO_2_ (< 3 wt%) Y_2_O_3_ (9 wt%) Al_2_O_3_+SiO_2_+ Others (< 2 wt%).	Dentsply Sirona, U.S. A
Zirconia Etchant Cloud System)	116,112 K	< 10% hydrofluoric acid < 2% thickening agent HF neutralizer gel.	Medifive, Korea
Zirconia primer	155,117 A	MDP Silane	Medifive, Korea
TheraCem^®^ Cement	2,300,110,087	Base: Calcium-based filler 20–50% Glass filler 30–50% Dimethacrylates 20–50% < 5% Ytterbium Fluoride Initiator 1–10% < 5% BisGMA Catalyst: < 30% 10-MDP Amorphous Silica 1–5%.	Bisco, U.S.A

### Sample size calculation

Sample size calculation was based on previous research investigating the effects of different surface conditioning protocols on the fracture load of monolithic zirconia partial coverage restorations [42]. Using G*Power program version 3.1.9.7 to calculate sample size based on an expected effect size of 0.88, a 2-tailed test, α error = 0.05, and power = 95.0%, the total calculated sample size will be 36 (at least 6 per subgroup). To provide additional specimens per subgroup and account for potential specimen exclusions during the experimental procedures, the sample size was increased to 8 specimens per subgroup, resulting in a total of 48 specimens (2 zirconia materials × 3 surface treatment protocols × 8 specimens).

### Samples’ preparation

Forty-eight sound human maxillary premolars of uniform size and morphology, extracted for periodontal or orthodontic purposes, were collected from the Oral Surgery Department at Mansoura University, Faculty of Dentistry. The chosen teeth were uniform in shape and size. The study eliminated teeth with cavities, fractures, or restorations. Selected teeth were disinfected for one week with 5.25% sodium hypochlorite household bleach diluted 1:10 [43]. To avoid dehydration during testing, the teeth were kept in distilled water at room temperature [44]. 

Teeth were selected based on their comparable shape and size (± 0.5 mm). The dimensions of each tooth at the cementoenamel junction were measured using a digital caliper (200 mm, 7.87 in, Total TMT322006 Digital Measuring Caliper 200 mm IP54, China). The selected teeth had mean dimensions of 8 ± 0.5 mm bucco-palatal, 7 ± 0.5 mm mesio-distal, and 4.4 ± 0.5 mm occluso-cervical. To enable the manipulation of the teeth, the roots of each tooth were vertically embedded in epoxy resin (Kemapoxy 150 3D, CMP International, Egypt). To ensure standardized preparation, all teeth were prepared by a single experienced prosthodontist, and a putty index was made for the whole tooth before beginning the preparation procedures. A Marathon-103 dental surveyor (Saeyang Co., Korea) was used for the preparation steps. The amount of reduction was assessed with a periodontal probe and a previously fabricated silicone index.

Occlusal preparation began with the creation of guiding grooves on the functional and non-functional cusps using a high-speed handpiece (NSK-Nakanishi International, Japan) with a depth cuter bur, and measured with the periodontal probe, then completed by a tapered round-end diamond stone (TR-12, MANI, Tochigi, Japan) and air-water coolant, resulting in a functional cusp of 1.5 mm and a non-functional cusp of 1 mm [4]. 

**Fig. 1:**
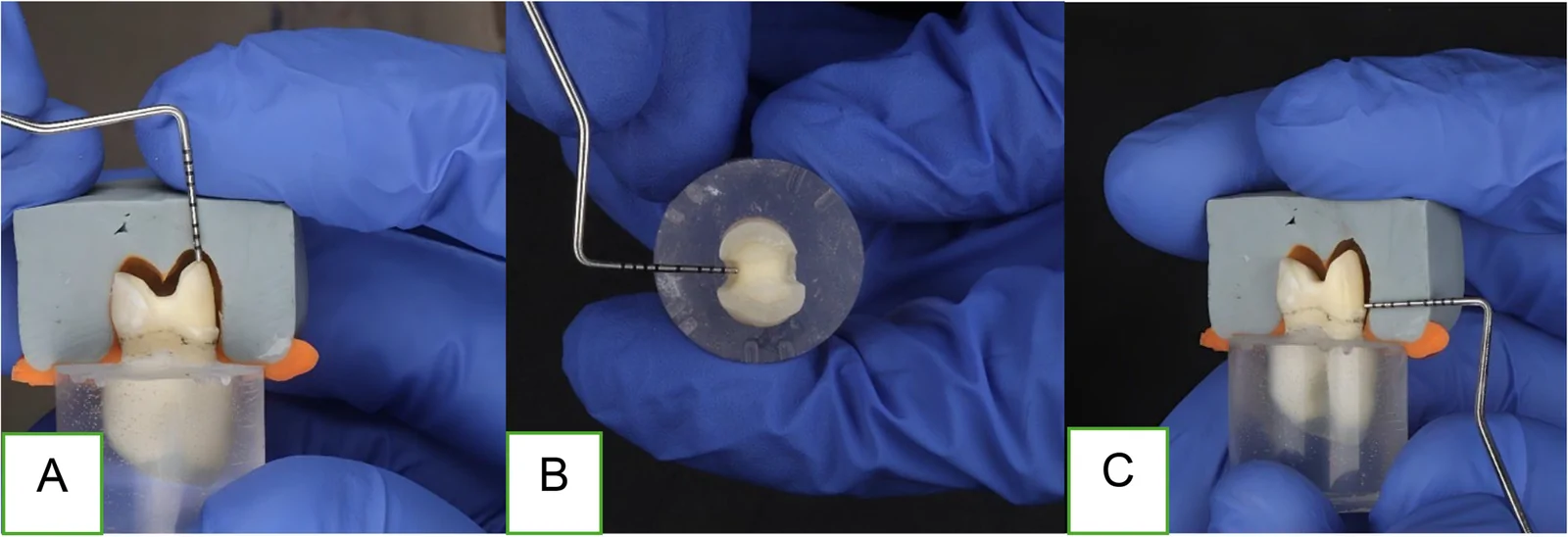
Checking the amount of preparation by the previously fabricated putty index

**Fig. 2:**
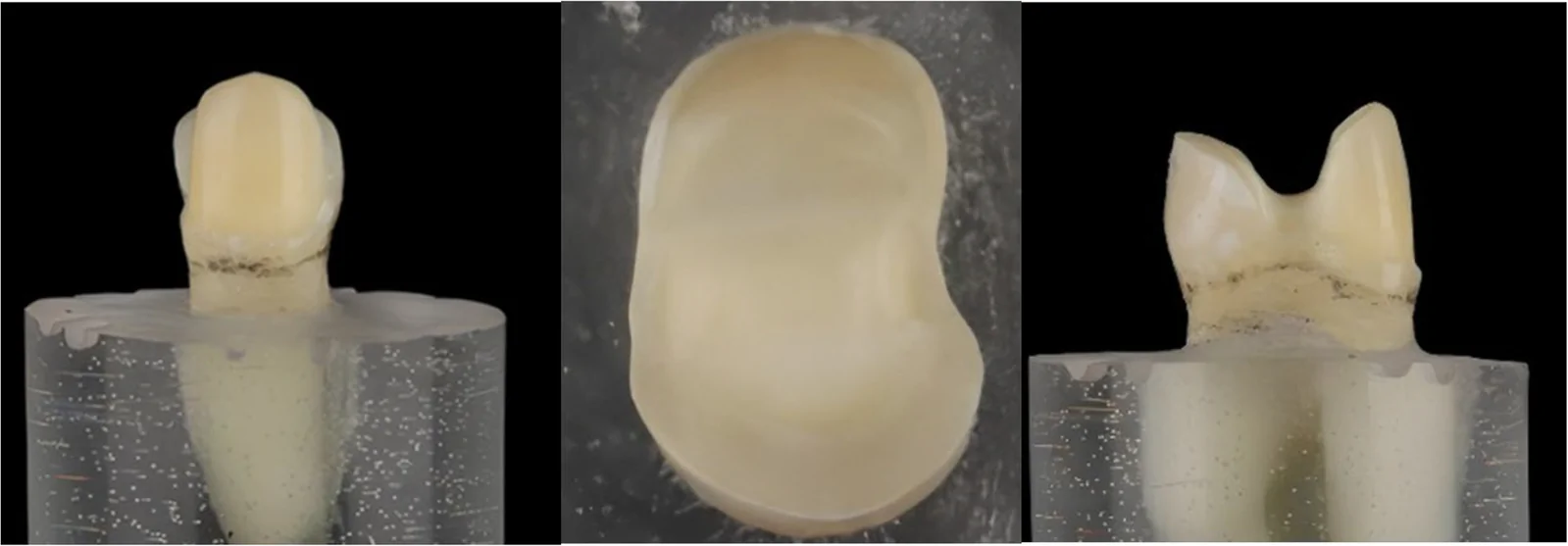
Buccal, occlusal, and proximal views of the finished preparation

For axial, pulpal floor, and proximal box preparation, a low-speed straight handpiece was attached to a dental surveyor with an external water-cooler. The pulpal floor cavity depth was 1 mm at the central groove, the occlusal cavity width was maintained at one-third the intercuspal distance, and each wall had a 3° taper using a CE-12 F diamond bur (MANI ISO 546/021) [45]. The mesiodistal width of the proximal box was set at 1 mm, and the gingival seat was positioned 1 mm above the cementoenamel junction [46]. With a depth-cutter bur and a taper-round end diamond stone, the preparation design was further expanded to the buccal surface, featuring a 0.5 mm chamfer finish line [4, 46, 47] Figs. 1 and 2.

After completing standardized tooth preparation, each specimen was assigned a unique identification number. A computer-generated randomization sequence (Randomization.com) was then used to allocate the specimens equally to the two zirconia material groups (*n* = 24 per material). Within each material group, the specimens were then allocated equally to the three surface-treatment subgroups (*n* = 8 per subgroup). The randomization sequence was generated before allocation, and specimen identification numbers were used throughout the experimental procedures to minimize allocation-related bias. 

### Study design

**Fig. 3:**
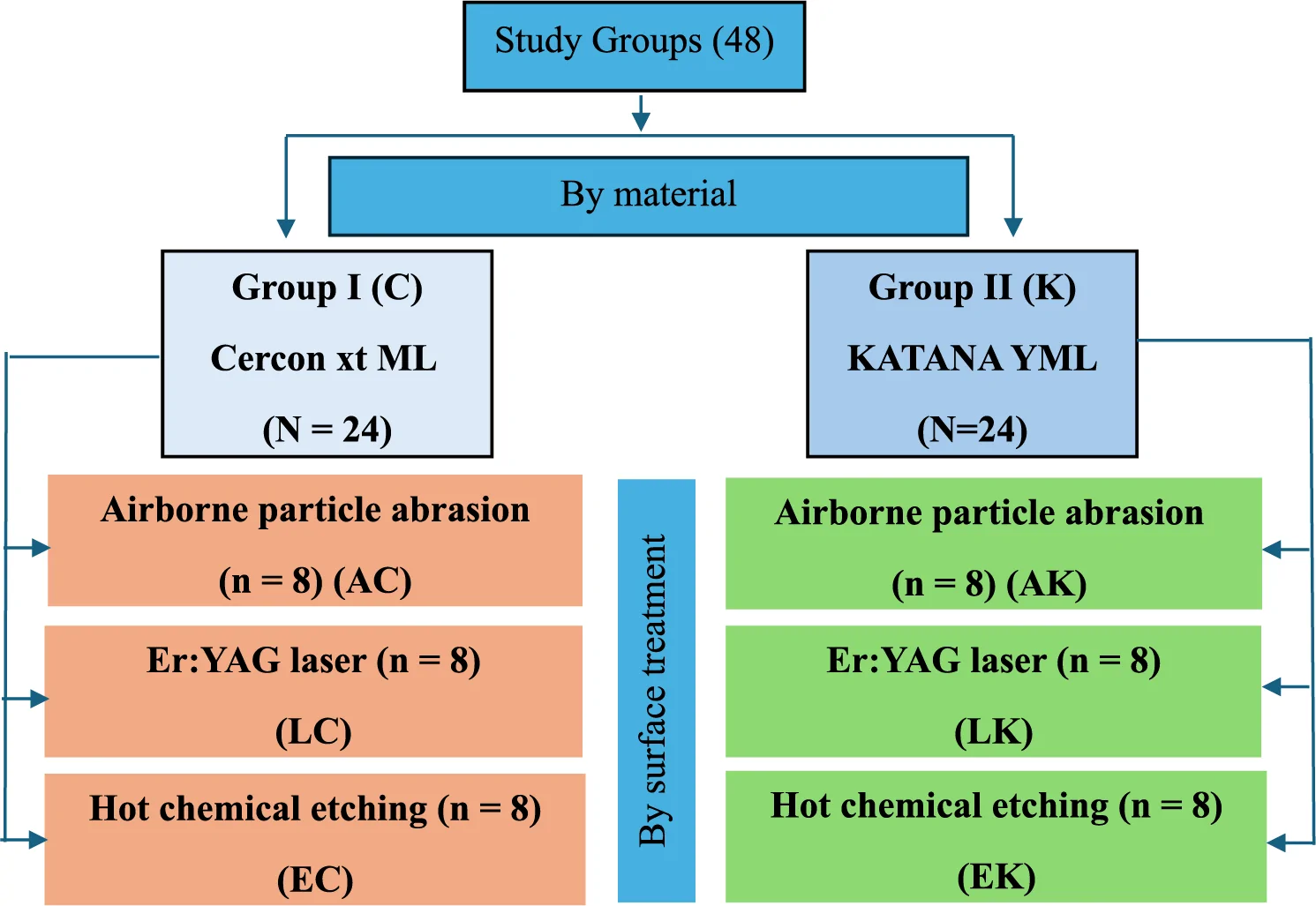
Showing study grouping

This in vitro study used a 2 × 3 factorial experimental design with two zirconia materials (Cercon xt ML and KATANA YML) and three surface treatment protocols (airborne particle abrasion, Er: YAG laser, and hot chemical etching). Forty-eight prepared maxillary premolars were equally allocated to the two zirconia material groups (*n* = 24 each), and each material group was further divided equally among the three surface treatment protocols (*n* = 8 per subgroup), resulting in six experimental subgroups. Fig 3. Study Groups (48).

### Fabrication of the restorations

**Fig. 4:**
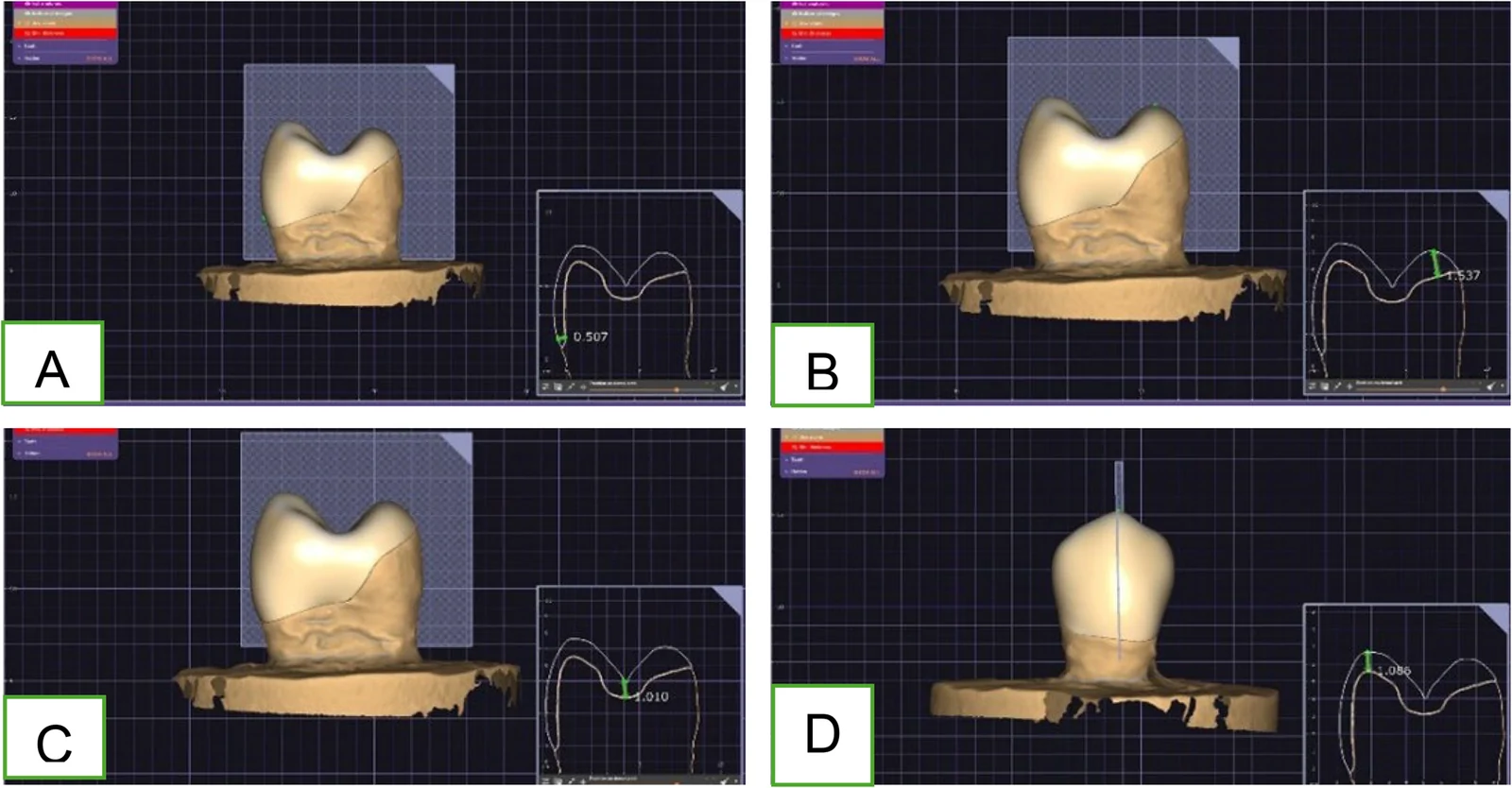
Verifying thicknesses of the restorations

Each prepared premolar was individually scanned by an extraoral optical scanner (DOF 3D Edge, Korea) to generate a separate virtual model. The software (Dental DB 2.2 Valletta, Exocad GmbH, Darmstadt, Germany) then used the scanning data to design the restoration. A tooth model of a maxillary first premolar was selected from the program library and modified slightly to conform to standard design requirements. All restorations were designed with standardized parameters. The occlusal thickness was set at 1.5 mm for functional cusp and 1 mm for non-functional cusp. The pulpal floor cavity depth was positioned 1 mm deep at the central groove, with each wall exhibiting a 3° taper. The mesiodistal width of the proximal box was 1 mm, and the gingival seat was positioned 1 mm above the cementoenamel junction. The buccal surface featured a 0.5 mm chamfer finish line. Each restoration was checked by the measurement tool in the Exocad program to ensure the same dimensions Fig. 4. 

The restorations were fabricated using a 5-axis milling machine (CERA MILL MOTION 2, Amann Girrbach AG, Herrschaftswiesen, Austria) from two types of zirconia blocks: KATANA zirconia YML (A2/D98.5 mm/T14mm) and Cercon xt ML (A2/D98.5 mm/T14mm). Following milling, the restorations were sintered in a sintering furnace (Tabeo-1/M/Zirkon-100, Germany) according to the manufacturer’s instructions. Subsequently, a final glaze (CERABIEN ZR FC, Kuraray Noritake Dental Inc., Japan) was applied, and the restorations were delivered into the Multimat Cube Press (Dentsply Sirona, Germany) furnace. After fabrication, all restorations were checked by a caliper to ensure uniform thickness. All sintered zirconia restorations were individually tried on their corresponding prepared premolar before cementation under magnification using dental loupes and with a sharp explorer to detect any marginal gaps.

### Surface treatment of zirconia restorations

#### Airborne particle abrasion subgroup (A)

The surface of the zirconia restoration was treated with µm aluminum oxide particles at 2 bar, with the nozzle 10 mm away at a 45-degree angle for 15 s, using a basic, classic fine sandblasting device from Renfert, Germany [28, 47]. The airborne particle abrasion procedure was performed under strictly standardized conditions for all specimens. A single sandblasting device was used, and all treatments were carried out by the same operator. Particle size, pressure, time, nozzle distance, and angulation were kept constant, and a continuous sweeping motion was used to ensure uniform surface treatment

#### Er: YAG laser subgroup (L)

The surface of zirconia restorations was exposed to radiation using an Er: YAG laser (Fidelis Plus III, Fotona, Slovenia). The following laser parameters were set: nm wavelength, 50 µs pulse duration, 10 Hz frequency, 200 mJ energy, and 2 W output power for 10 s. Fine air and water cooling were used at a 2/2 ratio during sample irradiation. The sapphire tip was positioned at a distance of around 0.5 mm perpendicular to the zirconia surface [29, 48, 49]. 

#### Hot chemical etching subgroup (E)

**Fig. 5:**
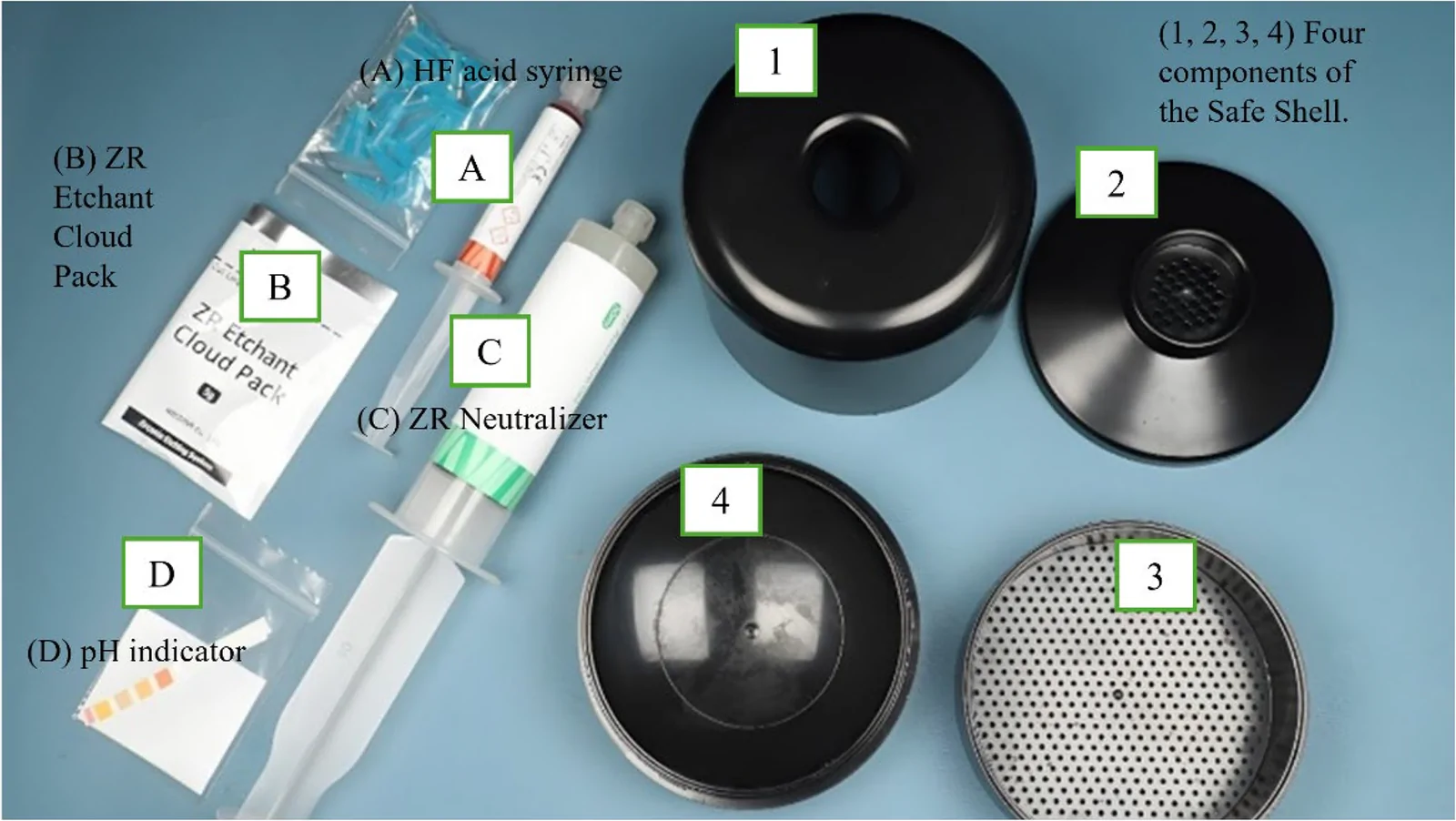
Preparing the components of the Zirconia Etchant Cloud System

**Fig. 6:**
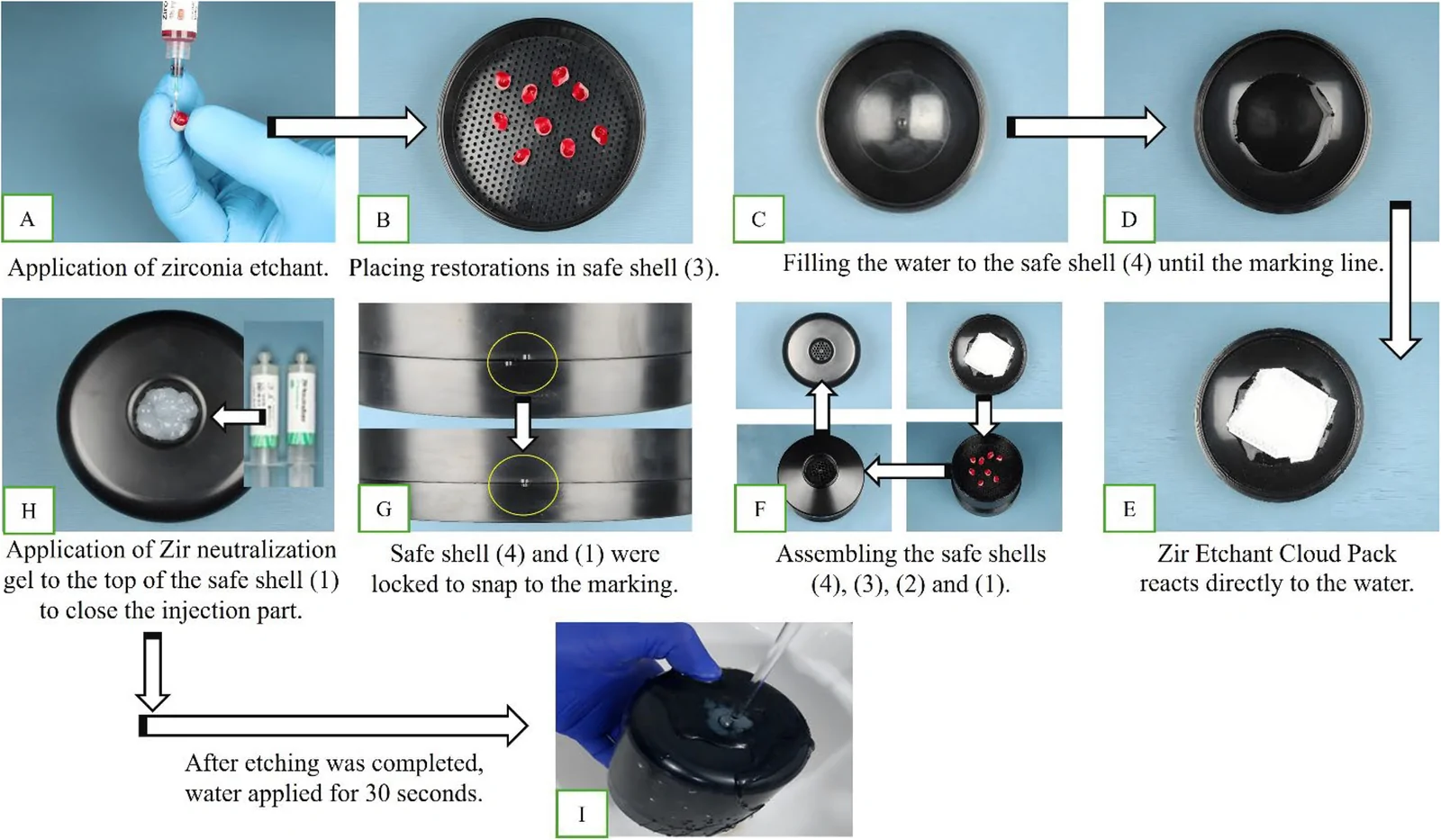
Steps of the hot chemical etching procedure

After preparing the components of the Zirconia Etchant Cloud System, low-concentration HF zirconia etchant (Ceramic Etchant 9, Medifive Co., Ltd., Incheon, Korea) was applied to the fitting surface of zirconia restorations, then the restorations were placed in the safe shell (3). Safe shell (4) was filled with water to the marking lines; the ZR Etchant Cloud Pack was placed in the center to be submerged in the water to begin the chemical reaction that is the source of heat generation. Then the components of the Zirconia Etchant Cloud System were assembled and locked with a triple locking design. An HF neutralizing agent (HF Neutralizer, Medifive Co., Ltd., Incheon, Korea) was placed on top of the safe shell (1) to close the injection part and to neutralize the hazardous chemicals and F ions released from the vessel’s upper section. This assembly was left for minutes to begin etching procedures. After minutes, the restorations were rinsed with water for 30 s at a sink. Safety protocols included clinician protective clothing and adequate site ventilation [18, 36, 39]. Figs. 5 and 6.

### Bonding of the zirconia restorations

The specimens were rinsed with water, cleaned in an ultrasonic bath containing 99% alcohol for three minutes, and dried to eliminate potential contamination introduced during surface treatment [50, 51]. Before applying resin cement, a zirconia primer containing MDP was applied to the intaglio surface of the restorations. Two layers were distributed across the entire fitting surface and rubbed for 30 s with a micro-brush. A gentle airflow was then applied for 3 to 5 s, as directed by the manufacturer.

Self-adhesive resin cement (TheraCem^®^ resin cement) was utilized for tooth cementation across all experimental groups. The cement was applied to the intaglio surface of each restoration, and the restorations were subsequently positioned on the prepared teeth under a constant static pressure of 10 N [52, 53]. The restorations were tack-cured with Elipar Deep Cure-S (3 M ESPE Dental, St. Paul, MN, USA). Excess cement was removed with a disposable scalpel. Each surface was subsequently treated for 20 s from four directions [53]. Following cementation, the bonded specimens were maintained at room temperature for one hour to ensure complete setting of the dual-cured resin cement and to minimize early polymerization stresses [18, 54]. A diamond polisher (DIACOMP PLUS RA-DCP-w11 m, EVE, Germany) removed residual cement from the margin of the restorations. For 24 h, cemented specimens were kept in distilled water at 37 °C [53]. 

### Artificial aging

Specimens were thermocycled using a thermal aging device (Thermo Scientific, Thermo Fisher Scientific Inc., Waltham, USA) to replicate intraoral conditions. Ten thousand cycles were used to simulate approximately one year of oral exposure. The protocol alternated temperatures between 5 °C and 55 °C, with a 30-second dwell time in each distilled water bath and a 5-second transfer interval [53, 55]. 

### Fracture resistance testing

Each specimen underwent a load-to-fracture test using a universal testing machine (Instron, USA, model 3345) under compressive loading. A 5 mm stainless steel ball was applied perpendicularly to the occlusal surface of the specimens at a crosshead speed of 1 mm/min. The fracture load for each specimen was recorded in Newtons (N). The occlusal surface of the specimen was covered with 0.5 mm-thick tin foil to prevent contact damage, improve the distribution of force, and prevent slippage of the load applicator [56]. 

### Failure mode pattern

A stereomicroscope (SZ61TR, Model SZ2-ILST) was employed to assess the fracture mode of each specimen at 15× magnification. Fracture modes were categorized as described in Table 2 [57]. Fracture mode evaluation was performed by a single blinded examiner who was unaware of the group allocation.

**Table 2:** Failure mode classification

Type	Description	Prognosis
I	Extensive crack formation within the ceramic.	Non-catastrophic/ repairable
II	Cohesive fracture within the ceramic or mixed failure (cohesive and adhesive) without dental involvement.	
III	Fracture within the tooth crown structure and the ceramic above the CEJ.	
IV	Longitudinal tooth and ceramic fracture involving the root.	Catastrophic/ non-repairable

Representative fractured specimens were examined with a scanning electron microscope (SEM) (JSM.6510LV, JEOL Ltd., Akishima, Tokyo, Japan)). Before imaging, a uniform 30 nm gold layer was sputtered onto the specimens for 180 s at 40 mA using an SPI Sputter Coater (SPI Module - Sputter Gold/Carbon Coater, SPI Supplies, Structure Probe, Inc., West Chester, PA USA).

### Statistical analysis

The statistical analysis was performed using IBM SPSS Statistics 26 software (IBM Corp., NY, USA). The normality of fracture resistance data was assessed using Kolmogorov–Smirnov test, which indicated no significant deviation from normality (*P* > 0.05). A two-way analysis of variance (ANOVA) was performed to evaluate the main effects of zirconia material and surface treatment protocol, as well as their interaction, on fracture resistance. When significant differences were detected, Bonferroni-adjusted pairwise comparisons were performed. One-way ANOVA was also performed to further assess the effect of surface treatment irrespective of zirconia material. Effect sizes for the two-way ANOVA were expressed as partial eta squared (ηp²) to quantify the magnitude of the effects. Statistical significance was set at α = 0.05.

## Results

**Fig. 7:**
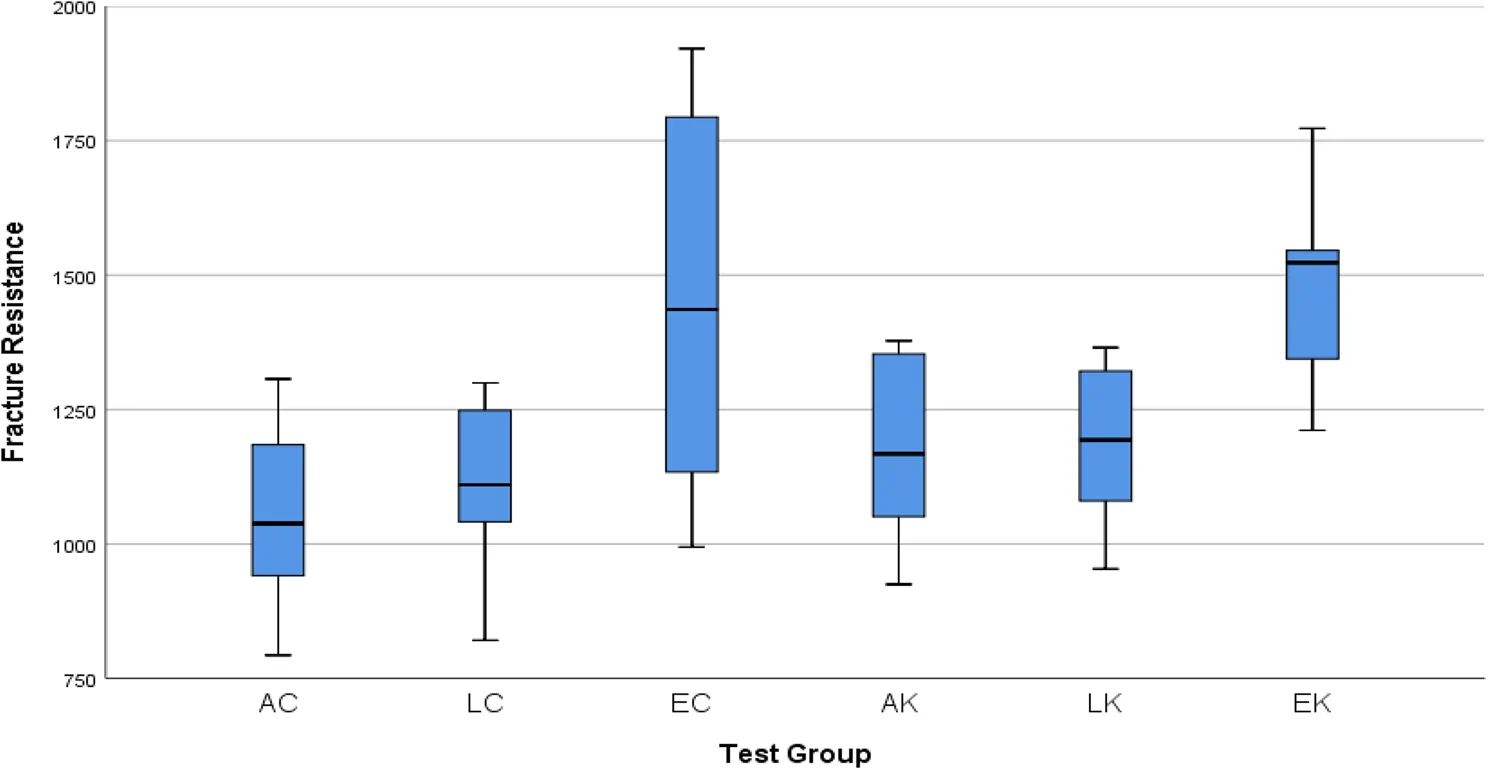
Hot chemical etching KATANA YML subgroup (EK) recorded the highest mean fracture resistance

**Fig. 8:**
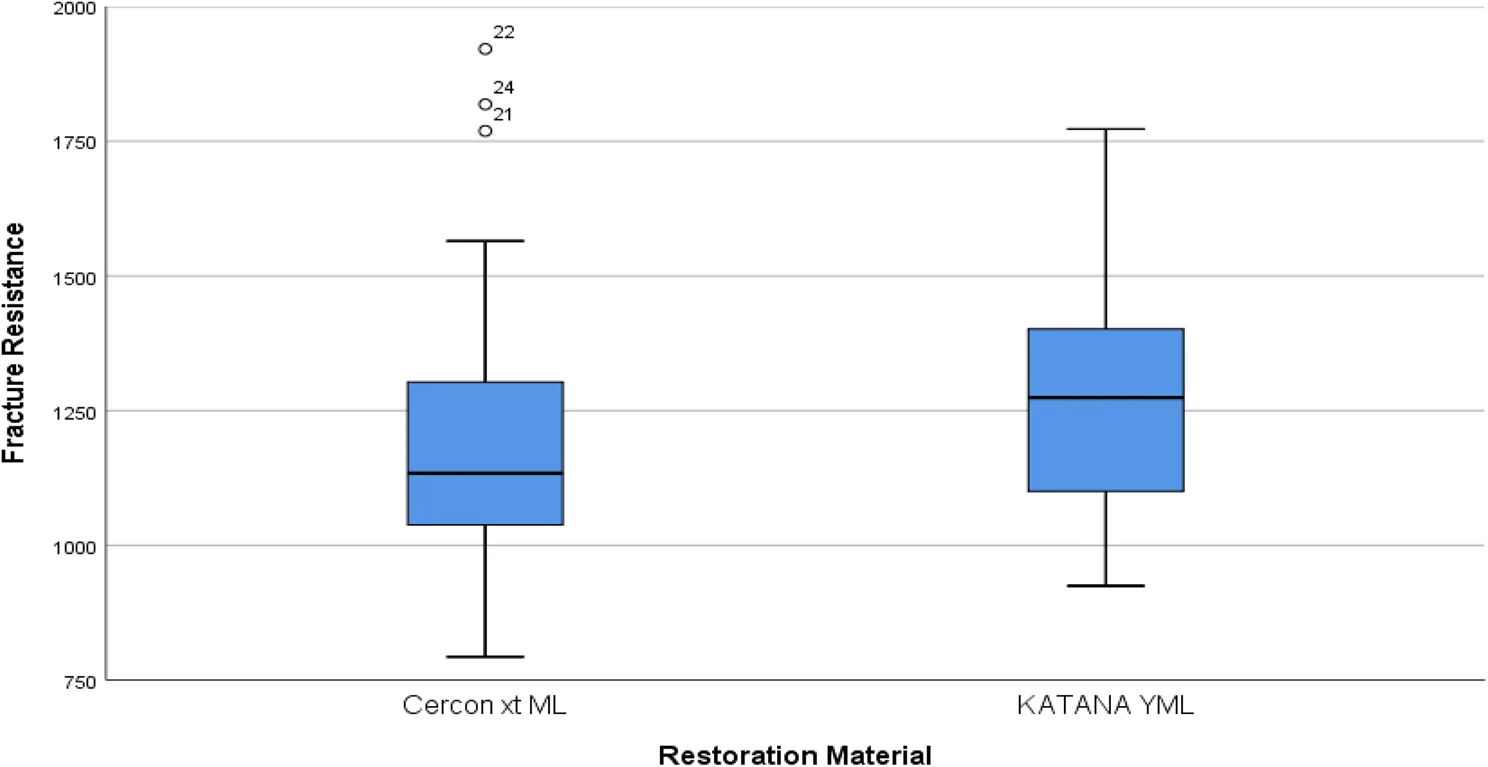
Mean fracture resistance values of the two zirconia materials

**Fig. 9:**
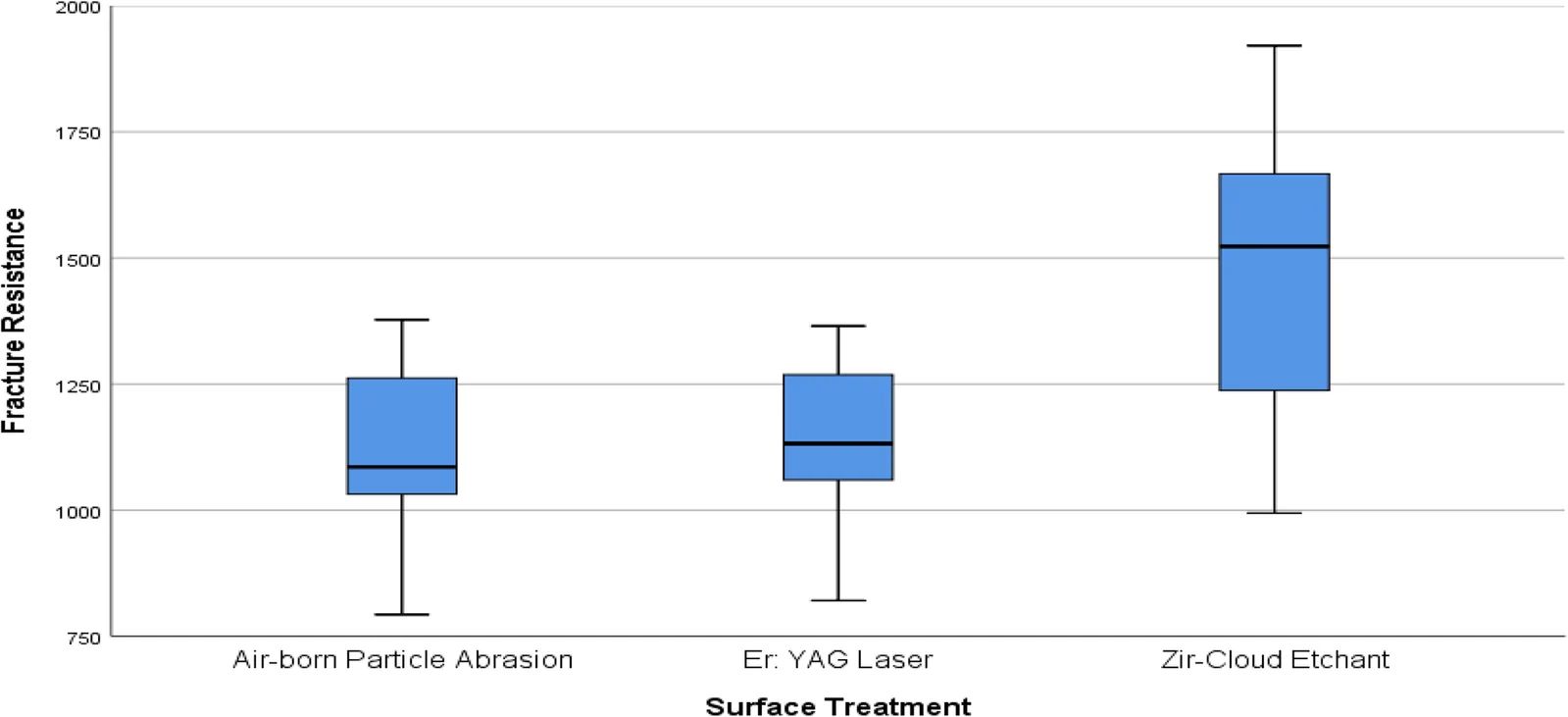
Hot chemical etching (Zir-Cloud Etchant) resulted in significantly higher fracture resistance than airborne particle abrasion and Er laser treatment

The boxplots presented in Figs. 7, 8 and 9 provide an overview of the fracture resistance values of zirconia material and surface treatment. The hot chemical etching KATANA YML subgroup (EK) showed the highest mean fracture resistance among all experimental subgroups. Although KATANA YML material showed numerically higher mean fracture resistance values than Cercon xt ML, the main effect of zirconia material was not statistically significant (*P* = 0.229). In contrast, surface treatment had a statistically significant effect on fracture resistance (*P* < 0.0001), with hot chemical etching producing higher fracture resistance than airborne particle abrasion and Er: YAG laser surface treatments. Means (SD) for all subgroups in Newtons are presented in Table 3.

**Table 3:** Means (SD) for all subgroups in Newtons

Test Groups	Fracture Resistance	
AC	N	8
	Mean	1053.33
	Std. Deviation	172.780
LC	N	8
	Mean	1114.98
	Std. Deviation	154.468
EC	N	8
	Mean	1455.36
	Std. Deviation	358.676
AK	N	8
	Mean	1180.68
	Std. Deviation	170.285
LK	N	8
	Mean	1188.575
	Std. Deviation	147.2550
EK	N	8
	Mean	1476.251
	Std. Deviation	177.336

**Fig. 10:**
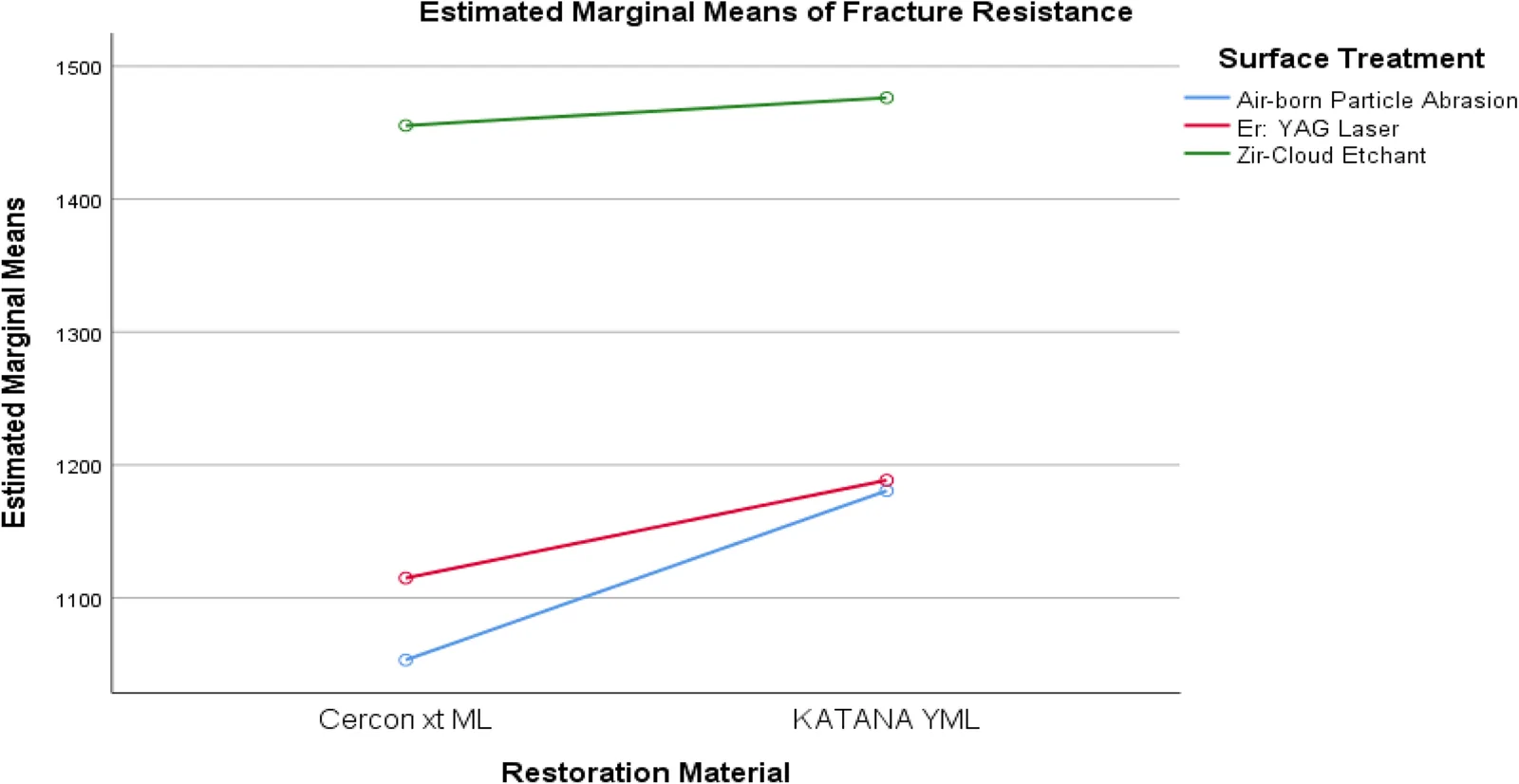
Estimated marginal means of fracture resistance for factors

Two-way ANOVA (Table 4) showed that surface treatment had a statistically significant effect on fracture resistance (*P*-value < 0.0001, ηp² = 0.0389), indicating a large effect. In contrast, the main effect of zirconia material was not statistically significant (*P* = 0.229, ηp² = 0.034). The material × surface treatment interaction was also not statistically significant (*P* = 0.774, ηp² = 0.012), indicating that the effect of surface treatment did not differ significantly by zirconia material (Fig. 10).

**Table 4:** Two-Way ANOVA of fracture resistance for material and surface treatment

Source	Type III Sum of Squares	df	Mean Square	F	Sig.	Partial η²	Observed Power^b^
Model	75654677.978^a^	6	12609112.996	286.039	< 0.0001		1.000
Material	65612.874	1	65612.874	1.488	0.229	0.034	0.222
Treatment	1181251.739	2	590625.869	13.398	< 0.0001	0.389	0.996
Material x Treatment	22674.767	2	11337.383	0.257	0.774	0.012	0.088
Error	1851437.951	42	44081.856				
Total	77506115.929	48					

The Two-Way ANOVA was followed by a Bonferroni Post Hoc Test for surface treatment, as indicated in Table 5, revealing that the hot chemical etching surface treatment exhibited a significantly higher fracture resistance than airborne particle abrasion (*P*-value < 0.0001) and Er: YAG Laser (*P*-value < 0.0001). No statistically significant difference was detected between airborne particle abrasion and Er laser treatment (*P* = 1.000).

**Table 5:** Post Hoc Test (Bonferroni test) for surface treatment based on the Two-way ANOVA test

Dependent Variable:	Fracture Resistance				
Bonferroni					
(I) Surface Treatment		Std. Error	Sig.	95% Confidence Interval	
				Lower Bound	Upper Bound
Er: YAG Laser	Airborne Particle Abrasion	74.23093692	1.000	-150.3340497	219.8804819
Hot chemical etching	Airborne Particle Abrasion	74.23093692	< 0.0001	163.6936873	533.9082189
	Er: YAG Laser	74.23093692	< 0.0001	128.9204711	499.1350027

The impact of surface treatment was subsequently assessed using a one-way ANOVA followed by a Bonferroni post hoc test. The post hoc test revealed that the hot-etched surface treatment differed significantly from both airborne particle abrasion (*P* < 0.0001) and Er: YAG laser (*P* < 0.0001) treatments, regardless of material type. Tables 6 and 7.

**Table 6:** The effect of surface treatment was evaluated using a one-way ANOVA test

ANOVA					
Fracture Resistance					
	Sum of Squares	df	Mean Square	F	Sig.
Between Groups	1181251.739	2	590625.869	13.702	< 0.0001
Within Groups	1939725.591	45	43105.013		
Total	3120977.330	47			

**Table 7:** Post Hoc Test (Bonferroni test) for surface treatment based on the One-way ANOVA test

Dependent Variable:	Fracture Resistance				
Bonferroni					
(I) Surface Treatment		Std. Error	Sig.	95% Confidence Interval	
				Lower Bound	Upper Bound
Er: YAG Laser	Airborne Particle Abrasion	73.40385986	1.000	-147.7660722	217.3125045
Hot chemical etching	Airborne Particle Abrasion	73.40385986	< 0.0001	166.2616647	531.3402414
	Er: YAG Laser	73.40385986	< 0.0001	131.4884486	496.5670253

### Failure pattern analysis

**Fig. 11:**
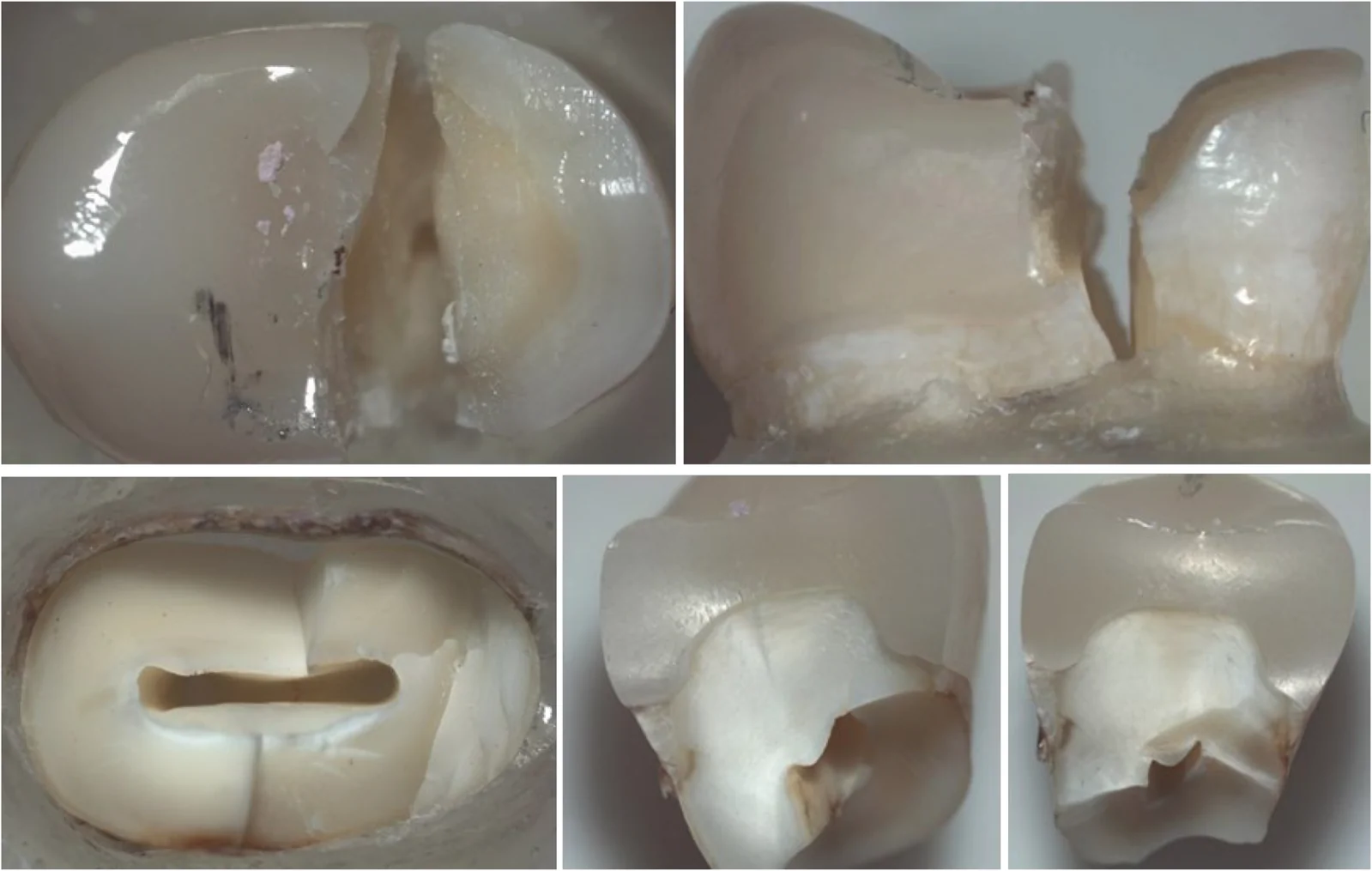
Type IV fracture

**Fig. 12:**
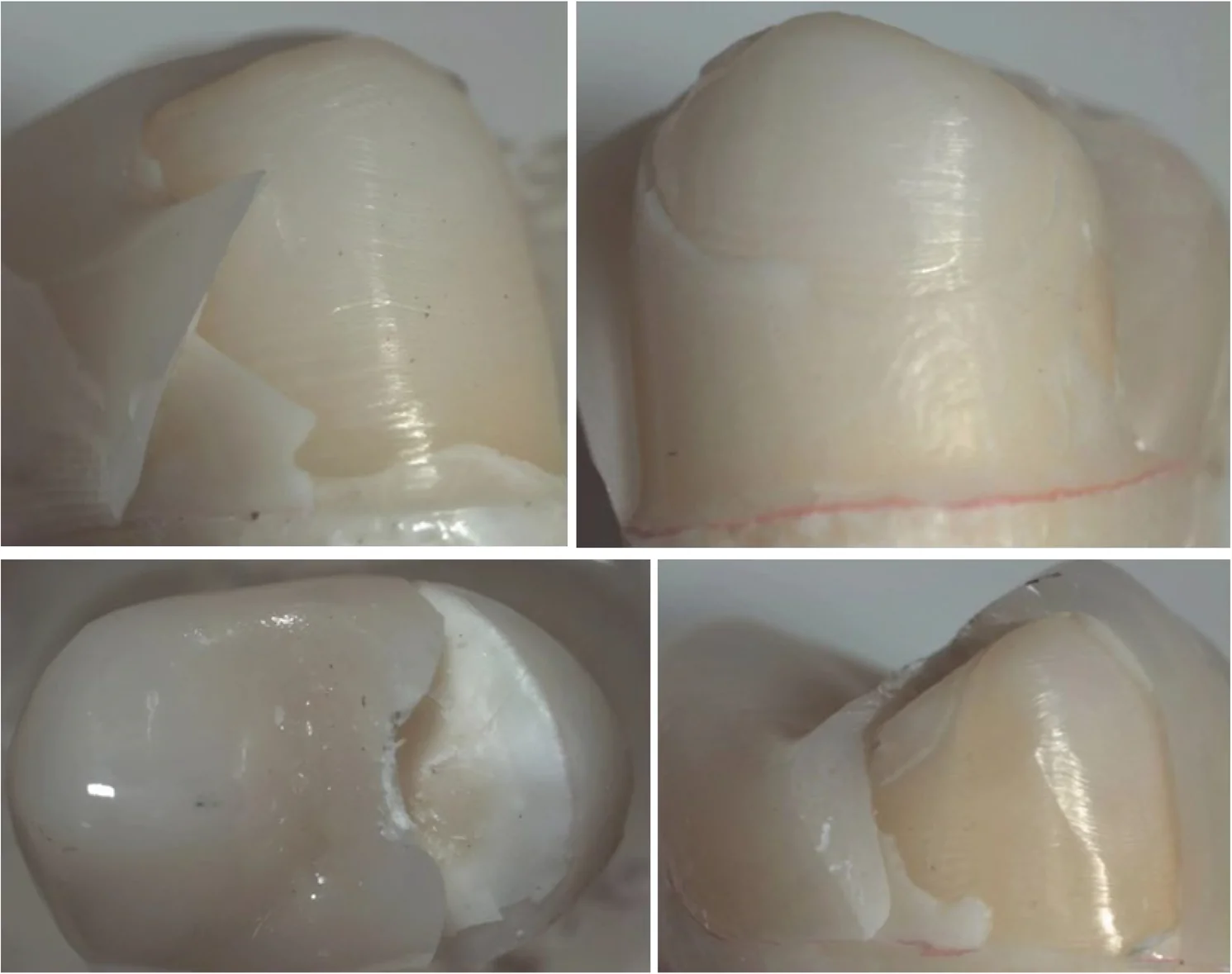
Type II fracture

Stereomicroscopic examination indicated that only type II and IV fracture patterns were observed in all specimens. All cracked specimens exhibiting the type IV fracture pattern featured a mesiodistal vertical fracture that bisected the restoration into two main sections. As shown in Fig. 11, most of these fractures progressed deeper into the tooth, leading to extensive root deterioration. Most specimens with the Type II fracture pattern showed fracture at the junction between the labial veneer and the occlusal surface (Fig. 12).

**Fig. 13:**
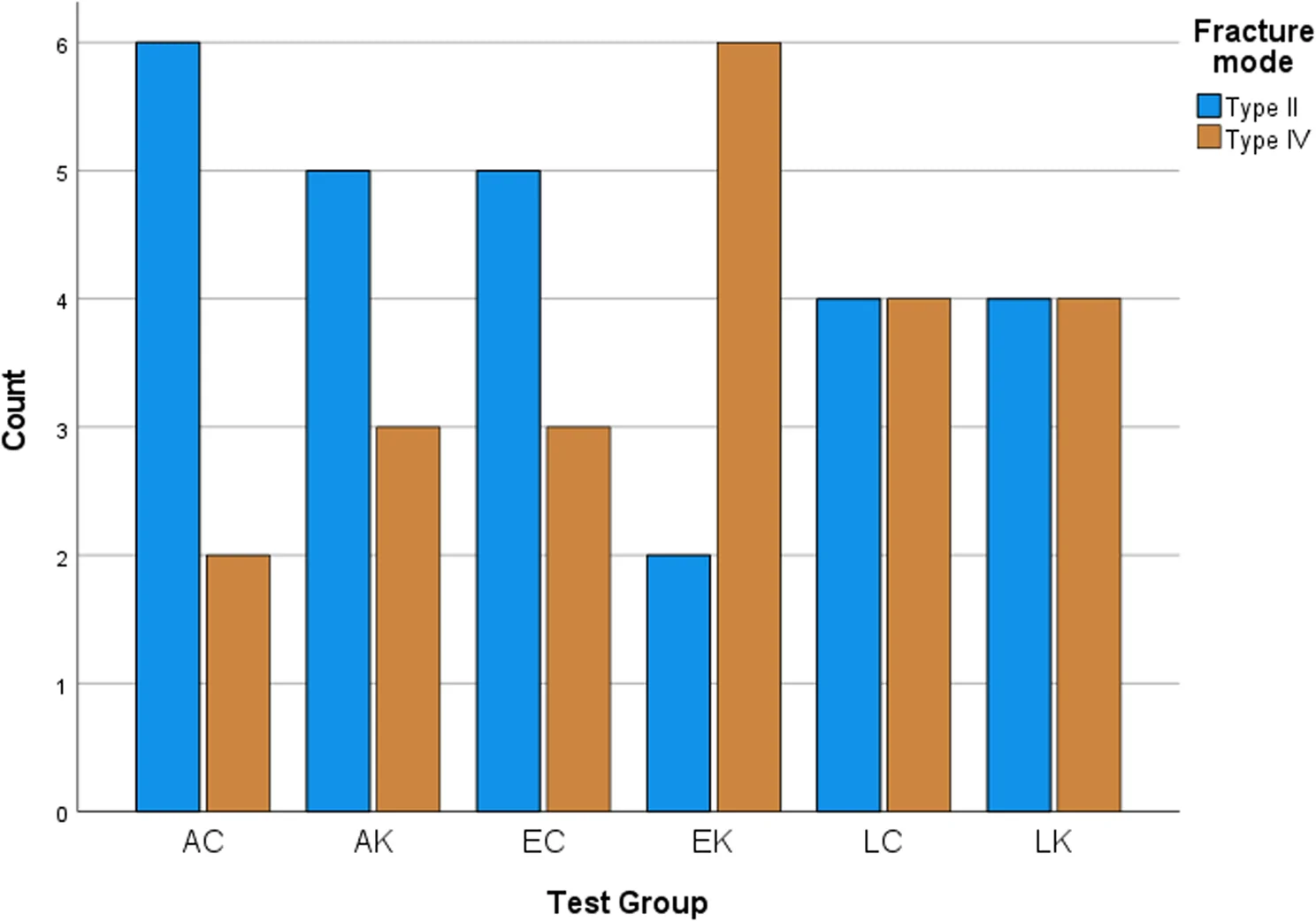
Distribution of fracture modes among all tested groups

**Fig. 14:**
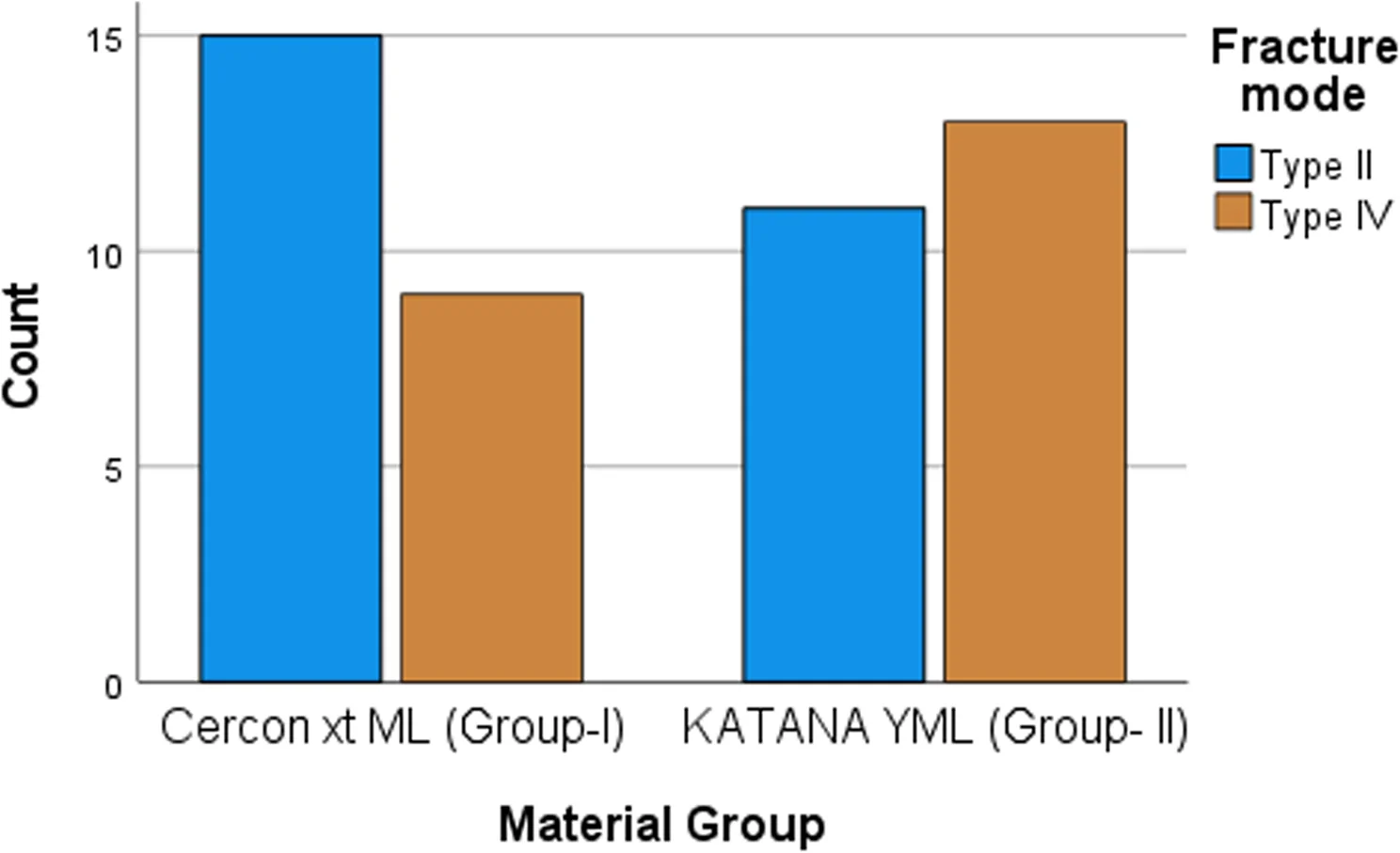
Distribution of Type II and Type IV fracture modes according to zirconia material

Only Type II and Type IV fracture patterns were observed among the tested specimens. Type IV fractures were observed more frequently in KATANA YML specimens than Cercon xt ML specimens; however, this difference was not statistically significant (*P* = 0.079). Similarly, the frequency of Type IV fractures did not differ significantly among the surface treatment groups (*P* = 0.070), although the etched group showed a numerically higher frequency (Figs. 13 and 14).

### Scanning electron microscope (SEM) examination

**Fig. 15:**
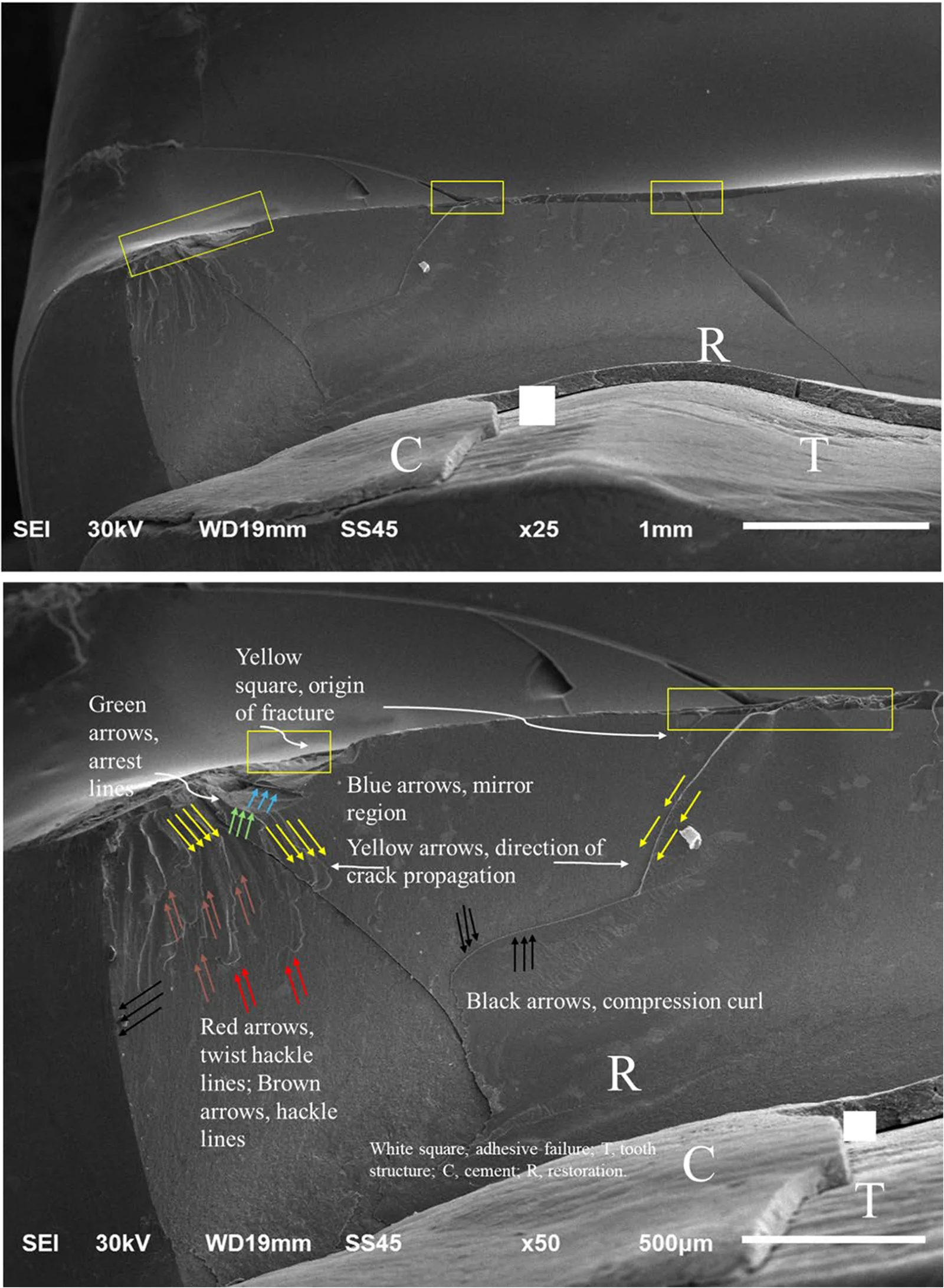
SEM analysis of Type II failure mode at 25x, 50x magnification

**Fig. 16:**
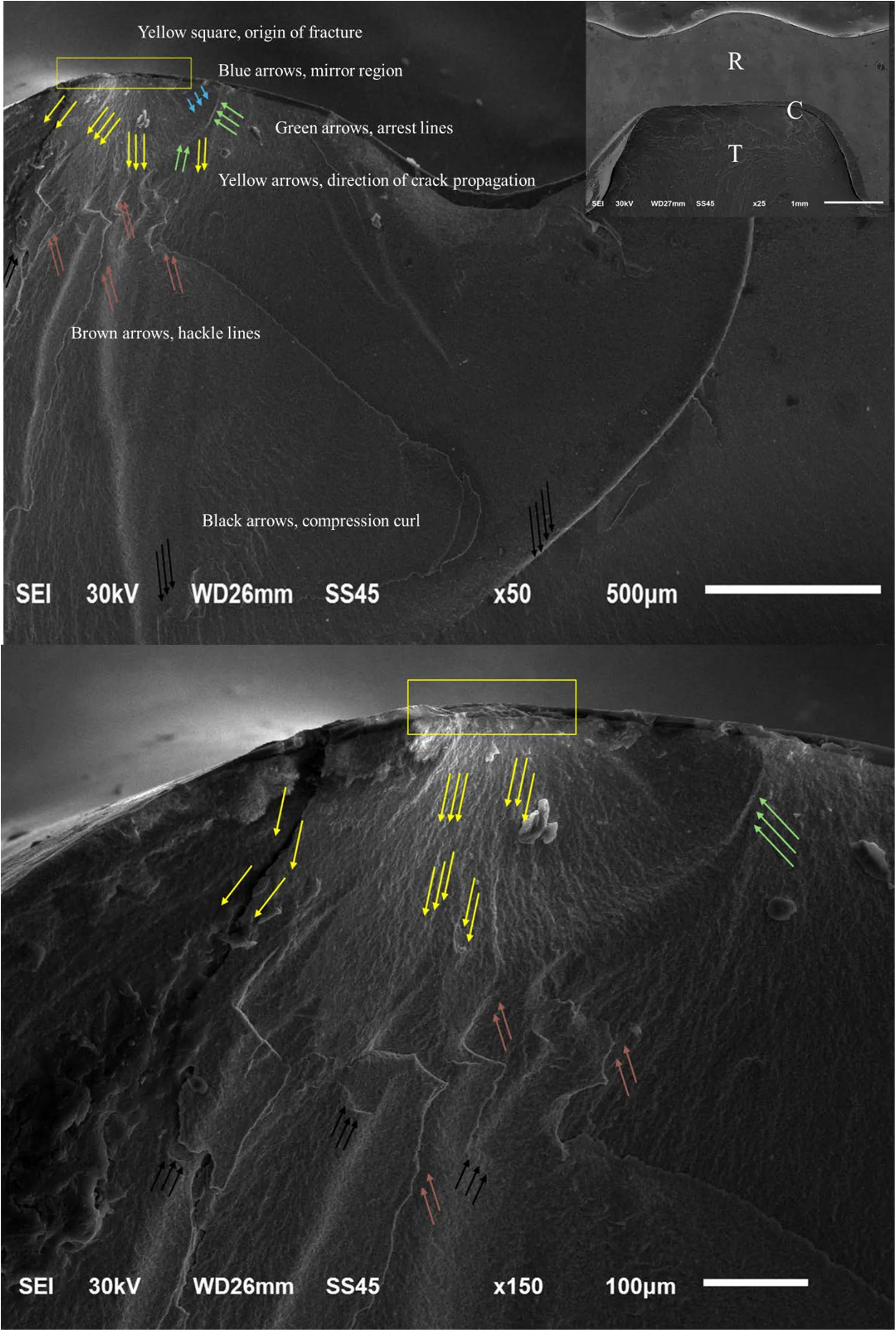
SEM analysis of Type IV failure mode at 25×, 50×, and 150× magnification. T, tooth structure; C, cement; R, restoration

Failure modes were further evaluated using scanning electron microscopy. Fractographic analysis identified the load origin as the point of failure initiation. The direction of crack propagation, as well as hackle lines and arrest lines, was thoroughly tracked (Figs. 15 and 16).

## Discussion

The results of the current study demonstrated that the fracture resistance of zirconia restorations was significantly influenced by the surface-treatment protocol used. Therefore, the null hypothesis stating that different surface-treatment protocols would not impact the fracture resistance of monolithic zirconia partial-coverage restorations is rejected.

This investigation utilized two types of monolithic zirconia with different compositions. The first material is Cercon xt ML, an extra-translucent zirconia. It is a multilayered, 5 mol% yttria-stabilized tetragonal polycrystalline zirconia (5YTZP). The second material is KATANA YML Zirconia, which consists of 3% yttria in the cervical part and 5% yttria in the incisal part, allowing for the evaluation of their performance under identical surface treatments.

Airborne particle abrasion settings must be optimized to achieve bond strength while minimizing side effects. To reduce the negative effects of airborne particle abrasion on zirconia restorations, this study used parameters supported by Zhang et al. (2021) [58], who found that 50 μm alumina particles at 0.2 MPa had minimal impact on the strength of transparent zirconia and maintained durable bonding with resin cement.

The Er: YAG laser was chosen for its ability to improve micromechanical retention and to produce a rougher surface on Y-TZP. Hatami et al. (2021) [30] identified this laser as the most effective method for achieving bond strength comparable to that achieved with sandblasting. The laser parameters in the present study were selected based on Zhu et al. (2022) [29], who found that high-power Er: YAG lasers can significantly damage Y-TZP crystals, leading to thermal degradation and reduced bond strength.

Hydrofluoric acid is frequently used in chemical etching methods for dental ceramics containing silica. However, zirconia does not undergo surface alteration when treated with hydrofluoric acid because of its glass-free composition [59]. Recent studies have investigated zirconia surface treatments using varying hydrofluoric acid (HF) concentrations, temperatures, and application durations. Elevated temperatures significantly influence molecular motion, as hot chemical etching enhances proton ionization in the acid solution. This process increases acidity and accelerates the removal of surface particles [36, 60–63]. 

Smielak et al. (2015) [64] reported that increasing both the etching duration and hydrofluoric acid (HF) concentration improves the surface quality of zirconia by enhancing surface roughness. Nevertheless, high-temperature, high-concentration etching solutions are challenging to manage and pose safety risks in dental offices or laboratories. Concentrations of 4% to 10% hydrofluoric acid (HF) are commonly used in dental clinics, and this range has been documented as safe for dental applications [36]. Consequently, in the current investigation, fracture resistance was evaluated after heat treatment using a 9% hydrofluoric acid (HF) solution in a triple-sealed vessel. HF gel was selected for its precise application and reduced flow, which enhanced safety compared to HF solution. An HF neutralizing agent was employed to mitigate hazardous compounds released from the upper section of the vessel. Safety protocols included the use of clinician protective clothing and sufficient site ventilation [18, 36, 39]. 

Self-adhesive resin cement was used to simplify the procedure and eliminate additional bonding steps [65]. However, a separate priming step was utilized in this study using an MDP-containing primer. This decision was based on evidence from previous studies that indicated that a separate priming step before cement application allows more effective and stable adsorption of MDP molecules onto zirconia. Such treatment has been shown to provide more reliable bonding than relying solely on the MDP incorporated in the self-adhesive resin cement. The use of MDP primer increases surface wettability and enhances chemical interaction between phosphate monomers and zirconia, resulting in improved resin adhesion to the zirconia surface [26, 66]. After cementation, the specimens underwent 10,000 thermal cycles to simulate one year of oral service [67]. This process may alter resin properties. Water absorption acts as a plasticizer, creating unsupported areas beneath restorations [53]. The occlusal surface of the specimen was covered with 0.5 mm-thick tin foil to prevent contact damage and improve the distribution of force. Additionally, the 5 mm stainless steel ball was carefully centered over the central fossa occlusal contact point of each specimen under visual inspection before load application. A controlled crosshead speed of 1 mm/min further minimizes the risk of slippage of the load applicator [56, 68]. 

The findings of this investigation revealed that the hot chemical etching subgroups (E) exhibited a superior fracture load value in both tested zirconia materials, followed by the Er: YAG laser (L), with the lowest mean fracture load value in the airborne particle abrasion subgroups (A).

The lowest mean fracture loads were observed in the airborne particle abrasion subgroups for both zirconia materials. This finding may be related to the response of highly translucent, multilayered zirconia to mechanical surface treatment. Airborne particle abrasion may produce surface irregularities, microdefects, and residual stresses that can act as sites for crack initiation and propagation under subsequent loading. Highly translucent zirconia contains a greater proportion of cubic phase and therefore has a reduced capacity for stress-induced transformation toughening compared with conventional 3Y-TZP [24]. 

The present study did not demonstrate a statistically significant main effect of zirconia material on fracture resistance (*P* = 0.229). Rather, the consistently lower mean values observed in the airborne particle abrasion subgroups suggested that surface treatment was significantly associated with fracture resistance, whereas material was not.

Previous studies have reported variable effects of airborne particle abrasion on highly translucent zirconia, indicating that its influence may depend on zirconia composition, yttria content, particle size, and blasting pressure [25]. McLaren et al. (2023) [24] demonstrated that the strength of a 4 mol% zirconia material exceeded that of 5 mol% zirconia when treated with airborne particle abrasion, as 5 mol% zirconia contains a higher cubic phase content than 4 mol% zirconia. Similarly, ALMutairi et al. (2021) [25] reported that 5 mol% zirconia material was negatively affected by air abrasion, as the increased yttria content of the 5Y-TZP resulted in a lower transformation of the tetragonal phase, and so a reduced release of compressive stresses and a decreased flexural strength.

Inokoshi et al. (2021) [23] showed that airborne particle abrasion enhanced the biaxial flexural strength of 4YPSZ and 5Y-PSZ but did not improve that of the highly translucent 6Y-PSZ zirconia. These findings indicated that the impact of abrasion on biaxial flexural strength is determined by the equilibrium between the accumulation of surface compressive stress and subsurface microcracks. Schabbach et al. (2023) [69] also reported that sandblasting had no significant impact on the phase composition or mechanical strength of multi-layered translucent zirconia. These findings indicated that the mechanical consequences of airborne particle abrasion in translucent zirconia were not uniform; therefore, in the present study, the lower fracture resistance observed in the airborne particle abrasion groups may be related to the surface treatment protocol, not the zirconia material.

The high mean fracture values in etched groups may result from the preserved integrity of the hot-etched zirconia surface. This can be explained by the different mechanisms of surface modification. Hot chemical etching has been reported to produce a uniformly micro-roughened and retentive surface topography through selective chemical dissolution of the zirconia surface without inducing impact forces, so this process did not induce subsurface microcracks, internal stress, or detrimental phase changes [36]. Also, the higher fracture load values in the etched group may be attributed to enhanced micromechanical interlocking with resin cement reported after hot chemical etching, together with the absence of mechanically induced damage that preserves the intrinsic strength of zirconia. This synergistic effect contributes to improved stress distribution at the adhesive interface and delays crack initiation during loading. These findings were consistent with Kim et al. (2022) [38], who found that hot-etched specimens exhibited significantly higher shear bond strength than sandblasted or untreated specimens. The authors attributed these findings to the observation that sandblasted samples displayed an irregular, uneven surface with strong cavities, while hot-etched samples showed sharply defined, small, homogeneous holes. Minimal tetragonal-to-monoclinic phase transformation was also observed in the hot-etched specimens.

Furthermore, Lv et al. (2023) [39] reported that an electron microscopy evaluation showed that the surface roughness acquired through sandblasting was distinct from that produced by acid etching. The micromechanical retention was improved more by the textured, granular surface generated by the etching process than by sandblasting, particularly when the etching was prolonged.

Previous studies have demonstrated that Er: YAG laser irradiation does not alter the crystalline characteristics of zirconia. The ratio of the monoclinic phase in the Er: YAG laser-treated samples was similar to that of the zirconia framework control groups. Liu et al. (2013) [70] clarified this using the laser’s water-cooling technique, which minimizes thermal degradation and maintains the monoclinic phase content within the structure. Çağlar and Yanıkoğlu (2016) [33] found that there were no significant variations in biaxial flexural strength values between the sandblasting and Er: YAG laser groups. However, SEM analysis revealed fractures on the zirconia surface at an energy intensity of 200 mJ. They explained that the cause of fractures in the Y-TZP surface may be related to the duration of application.

Conversely, Da Silva et al. (2021) [48] found that Er: YAG laser treatment significantly reduced the biaxial flexural strength of zirconia. These findings suggested that laser irradiation, particularly at extended pulse durations, can damage the zirconia surface and compromise its mechanical properties. The decrease in biaxial flexural strength highlighted the need to optimize laser parameters to preserve the integrity of zirconia restorations.

No significant interaction was identified between zirconia material and surface treatment (*P* = 0.774), indicating no statistical evidence that the effect of the different surface treatment protocols differed by zirconia material. Although the two zirconia materials have different compositions and microstructure characteristics, those differences did not produce a statistically significant difference in fracture resistance under the experimental conditions [71, 72]. Both zirconia materials demonstrated fracture loads that exceeded the maximum reported physiological masticatory forces, supporting their potential suitability for posterior clinical applications. Reports indicated that the mean masticatory force in the anterior region is 200 N, in the posterior region is 350 N, and for parafunctional habits is 1000 N. The maximum clenching and bruxism forces in patients might attain 1221 N [73].

Regarding failure mode analysis, no statistically significant differences were observed between the zirconia material or among surface treatment groups. The observed patterns may reflect specimen-to-specimen variability under the experimental loading conditions rather than a specific effect of the tested material or surface treatment protocol.

The strengths of this study included evaluating clinically relevant zirconia partial coverage restorations, which reflect a minimally invasive restoration approach. It provided a direct comparison of three different surface-conditioning protocols proposed for zirconia restoration under a standardized methodology. To our knowledge, this study evaluated a relatively novel surface-conditioning method (hot chemical etching) on the mechanical behaviour of translucent zirconia, contributing to the limited existing evidence on this technique.

### Limitations

1. It was nearly impossible to achieve ideal uniformity in natural teeth in terms of dimensions and biological variability.
2. The lack of mechanical cyclic loading and long-term water storage constituted a major limitation of this study and affected the extrapolation of the results to long-term clinical performance. The study did not reproduce the repeated, submaximal and variable loading experienced clinically during mastication and parafunctional activity.
3. The absence of profilometry, X-ray diffraction, and SEM analysis limited the correlation of surface roughness, phase transformation, and microstructural changes with the observed fracture resistance. Additionally, the present study did not include layer-specific analysis to determine the site of fracture initiation.
4. This study did not include an untreated zirconia control group. Consequently, the design allowed comparison of relative fracture resistance among airborne particle abrasion, Er: YAG laser treatment, and hot chemical etching, but did not permit determination of the absolute effect of each conditioning protocol on an untreated surface.
5. The roots were embedded in rigid epoxy resin without the use of a viscoelastic material to mimic the periodontal ligament.
6. Only a uniaxial load was applied; therefore, clinically occurring lateral forces were not reproduced.
7. Regarding fracture mode evaluation, examiner calibration and intra-examiner reliability assessment were not conducted.

## Conclusions

Considering the limitations inherent to this in vitro study, the following conclusions can be drawn:

1. Surface treatment protocol significantly affected the fracture resistance of the tested zirconia restorations.
2. The fracture resistance of the tested translucent zirconia falls within the clinically accepted range. Regarding the fracture test, hot chemical etching resulted in higher fracture resistance than airborne particle abrasion or Er: YAG laser surface treatment.
3. No statistically significant difference in fracture resistance was observed between the two zirconia materials.

## Data Availability

The corresponding author can provide the data sets used and/or analyzed in this study upon reasonable request.

## Abbreviations

Er: YAG: Erbium-doped yttrium aluminium garnet laser
Al_2_O_3_: Aluminum Oxide
HF: Hydrofluoric Acid
N: Newton
SEM: Scanning electron microscope
YML: Yttria multilayer
MDP: Methacryloyloxyidecyl-Dihydrogenphosphate
